# Supplementary material for: A high-resolution mRNA expression time course of embryonic development in zebrafish
Source: eLife. 2017 Nov 16;6:e30860. doi: 10.7554/eLife.30860 (PMC5690287; doi:10.7554/eLife.30860)
Supplement: Supplementary file 6. [file elife-30860-supp6.zip › biolayout-clusters-files/Cluster219-genes.html]

Cluster219


# Cluster219: Genes

| Ensembl ID | Gene Name | Chr | Start | End | Biotype |
| --- | --- | --- | --- | --- | --- |
| ENSDARG00000076118 | cdip1 | 3 | 48435526 | 48458053 | protein\_coding |
| ENSDARG00000059804 | fam134c | 3 | 36941907 | 36966015 | protein\_coding |
| ENSDARG00000036308 | nod1 | 16 | 54024936 | 54039892 | protein\_coding |
| ENSDARG00000074310 | rbfox3l | 3 | 8445844 | 8766102 | protein\_coding |
| ENSDARG00000055974 | tpmt.1 | 19 | 47698691 | 47707061 | protein\_coding |
| ENSDARG00000089517 | zgc:92594 | 7 | 73605973 | 73613574 | protein\_coding |
